# From imbalance to impairment: the central role of reactive oxygen species in oxidative stress-induced disorders and therapeutic exploration

**DOI:** 10.3389/fphar.2023.1269581

**Published:** 2023-10-18

**Authors:** Sheryar Afzal, Aimi Syamima Abdul Manap, Ali Attiq, Ibrahim Albokhadaim, Mahmoud Kandeel, Sameer M. Alhojaily

**Affiliations:** ^1^ Department of Biomedical Sciences, College of Veterinary Medicine, King Faisal University, Al-Ahsa, Saudi Arabia; ^2^ Discipline of Pharmacology, School of Pharmaceutical Sciences, Universiti Sains Malaysia, Minden, Malaysia; ^3^ Department of Pharmacology, Faculty of Veterinary Medicine, Kafrelsheikh University, Kafrelsheikh, Egypt

**Keywords:** oxidative stress, epigenetic marks, reactive oxygen species, animal models, drug discovery

## Abstract

Increased production and buildup of reactive oxygen species (ROS) can lead to various health issues, including metabolic problems, cancers, and neurological conditions. Our bodies counteract ROS with biological antioxidants such as SOD, CAT, and GPx, which help prevent cellular damage. However, if there is an imbalance between ROS and these antioxidants, it can result in oxidative stress. This can cause genetic and epigenetic changes at the molecular level. This review delves into how ROS plays a role in disorders caused by oxidative stress. We also look at animal models used for researching ROS pathways. This study offers insights into the mechanism, pathology, epigenetic changes, and animal models to assist in drug development and disease understanding.

## 1 Background

The inability of the reactive oxygen species (ROS) scavenger to control the overproduction of oxidants leads to oxidative stress (Lushchak and Storey, 2021). The majority of vertebrates use the oxidation of oxygen molecules to create energy. At the tip of the respiratory chain, in the mitochondria, where the final step in the reduction of oxygen molecules is taken, water is produced. However, there may be a partial reduction in oxygen molecules, resulting in the production of reactive oxidant intermediates including superoxide anion (O2•-) and hydroxyl radicals (HO•), which trigger subsequent oxidation (Annarapu et al., 2021). Furthermore, mitochondrial origins, nicotinamide adenine dinucleotide phosphate (NADPH) oxidases, xanthine oxidase (XO), defective endothelial nitric oxide synthase (eNOS), and enzymes implicated in arachidonic acid metabolism are the principal sources of ROS inside the arterial lining (Table 1).

**TABLE 1:** Types of ROS and Sources in the vascular wall.

Reactive oxygen species (ROS)	Sources in the vascular wall
Superoxide anion radical (O -)	NADPH oxidase
Hydroxyl radical (HO-)	Xanthine oxidase
Hydrogen peroxide (H2O2)	Uncoupled nitric oxide synthase (eNOS)
Nitric oxide (NO)	Cyclooxygenase and lipoxygenase
Peroxynitrite (ONOO-)	Mitochondrial respiratory chain Hypochlorous acid (HOCI)

Glutathione peroxidase (GPx), superoxide dismutase (SOD), and glutathione (GSH) are antioxidant enzymes that protect mitochondria from ROS (Bhatia and Sharma, 2021). This non-protein antioxidant is well-known for protecting cells from the damaging effects of oxidative stress. Some antioxidant enzymes involved in detoxication methods, such as glutathione peroxidase (GPx-), glutathione transferase, dehydroascorbate, and reductase, require GSH as a cofactor. Glutathione is more frequently found in its reduced form than in its oxidized form (GSSG) in physiological settings. It is a crucial part of the antioxidant system and provides defense against oxidative damage as well as aids in cell detoxification activities. The nucleophilic and reducing characteristics of the naturally occurring tripeptide GSH play a crucial role in metabolic processes. It plays a role in the movement of amino acids across cell membranes. According to Ďuračková and Gvozdjáková, 2008, GSH directly scavenges singlet oxygen (^1^O_2_) and hydroxyl radicals (HO•) (Ďuračková and Gvozdjáková, 2008). Vitamins C and E are converted back into their active forms by GSH. According to Schafer and Buettner (2001), the ratio of GSH to GSSG reveals the redox status of cell (Schafer and Buettner, 2001) Therefore, the reduced levels of GSH peroxidase and GSH-S-transferase determine the defense mechanism against ROS (Aoyama, 2021).

Because of its capacity to convert extremely reactive superoxide radicals into hydrogen peroxide (H2O2) and oxygen, SOD is thought to be the first line of defense against ROS (Marwicka and Zięba, 2021). The GPx can lower ROS levels by acting as a catalyst in the reduction of hydrogen peroxide, which transforms to water or alcohol (Andrés et al., 2022) Lowering GSH activity is another way. It will generate oxidized GSH in the presence of GSH, considerably safeguarding a multicomponent antioxidant defense mechanism within the cell. ROS can be formed endogenously or exogenously and are affected by their surroundings.

**FIGURE 1:**
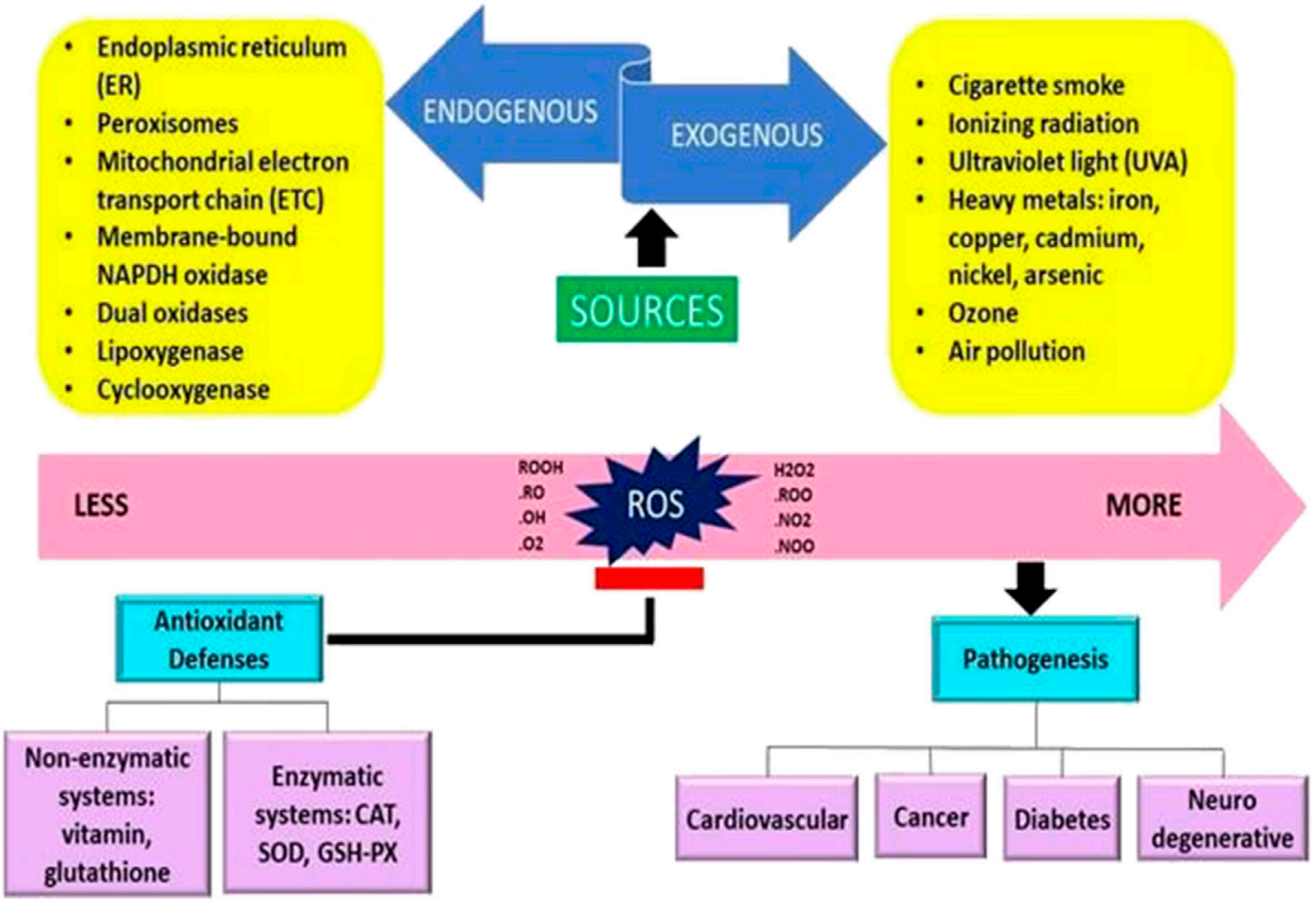
The origins of free radicals and their impact on the human body are presented schematically (Adaptation and Modification from Sharifi-Rad et al., 2020).

Free radicals are often created internally by intracellular metabolism when the antioxidant systems are overworked and externally as a consequence of the effect of things like pollution and cigarette smoke (Sharifi-Rad et al., 2020). Extracellularly, the upsurge in the ROS production in cells may be caused by a variety of environmental triggers, including exposure to cigarette smoke, UV radiation, heavy metal ions, ozone, allergens, drugs or toxins, pollutants, pesticides, or insecticides (Dos Santos et al., 2018; Mahajan et al., 2019) (Figure 1).

ROS produced by heavy metals can induce the ROS by direct or indirect reaction (Paithankar et al., 2021). For the indirect method, the heavy metal is required to undergo a reaction to produce the free radicals. At the same time, free radicals can be produced by directly acting between heavy metal and cellular compounds. Heavy metals directly cause an increase in lipid peroxidation and GPx-levels in the target area (brain tissue). This may cause the brain to be susceptible to damage due to oxidative stress (Paithankar et al., 2021; Raj et al., 2021). When NADPH electrons are transferred to molecular oxygen during the cellular respiration process, endogenous sources such as NADPH oxidase boost the formation of radical superoxide (Costa et al., 2021). Because of the anionic characteristics of radical superoxide, it can diffuse across a lipid membrane, leading to a rise in hydrogen peroxide and hydroxyl radical (HO•) by lowering the interior of cells (Costa et al., 2021; Tian et al., 2021). Among the disorders connected to mitochondrial ROS (mtROS) formation include inflammation, cancer, neurological illness, diabetes, and aging (Cabello-Verrugio et al., 2018) Cancers can have elevated ROS levels and mitochondrial malfunction, resulting in oxidative damage to cellular components. ROS generation in the cell has the potential to harm lipids, proteins, and DNA. ROS may damage the lipid membrane by increasing its permeability and fluidity (Hayes et al., 2020). This comprehensive research aims to provide light on the most current results about the health implications of oxidative stress, potential biomarkers, and the many animal models employed. It also provides sufficient baseline evidence for therapeutic antioxidant intervention.

## 2 Ros and normal cellular functions

The role of ROS has gained greater recognition in the past 4 decades. Oxidative stress is an imbalance that develops when the body produces more free radicals than it can effectively neutralize (Mittler, 2017). Oxidative stress arises from an elevation in ROS levels and has been associated with numerous adverse effects on cells’ balance, composition, and operation. However, the perception of ROS has evolved from being considered harmful byproducts of oxidative stress to beneficial agents in regulating normal cellular processes, known as redox signaling. Redox biology examines the role of elevated ROS as messengers in regulating physiological functions, and how their interplay can influence both health and disease conditions. Oxidative stress is characterized by excessive ROS levels that harm DNA, proteins, or lipids. On the other hand, redox biology deals with minor increases in ROS that trigger signaling routes to initiate biological responses (Schieber and Chandel, 2014).

When appropriately controlled, ROS plays a vital role in cellular activities. Cells naturally regulate the production of ROS. However, when ROS levels increase, they can become harmful, leading to oxidative stress and disease. Various cellular and biochemical studies have detailed the chemical properties of ROS oxidants. Yet, it is not always clear how these findings relate to chemical reactions that involve redox changes (Juan et al., 2021).

In addition to inducing irreversible oxidative modification of proteins (protein oxidation and peroxidation), lipids, and glycans (advanced lipoxidation and glycation end products), excessive ROS not only causes genomic mutations but also promotes disease or cell death when unchecked by protective antioxidant systems. Contrarily, low-level local ROS are crucial for regulating essential transcription factors such as NF-B/I-B, Nrf2/KEAP1, AP-1, p53, HIF-1, and PTK/PTP, as well as for maintaining cellular homeostasis (MAPK/ERK, PTK/PTP, PI3K-AKT-mTOR). As a result, ROS can influence a range of cellular processes, including apoptosis, migration, differentiation, and proliferation (Checa and Aran, 2020).

Several studies provide evidence that ROS, particularly O2 and H2O2, act as signaling molecules within cells (Franchina et al., 2018; Weinberg et al., 2019; Checa and Aran, 2020) In bacteria, the transcription factors SoxR and SoxS mediate O2 responses, while OxyR activates various genes induced by hydrogen peroxide (H2O2) (Rhen, 2019; Vazulka et al., 2022) Oxidative stress responses are more sophisticated in higher eukaryotes, including animals and plants, and are controlled by several regulators (Mullineaux et al., 2018). Cysteinyl thiol redox cycling is required for the formation of protein-protein and protein-DNA interactions, which characterize many components of signal transduction pathways (Hopkins and Neumann, 2019; Huang and Li, 2020) Proteins such as nuclear factor kB NF-kB) and activator protein-1 (AP-1) promote proliferation, differentiation, and morphogenesis and can be triggered by a variety of stimuli (Kirtonia et al., 2020) This stimulation can happen via a common mechanism, such as ROS generation.

**FIGURE 2:**
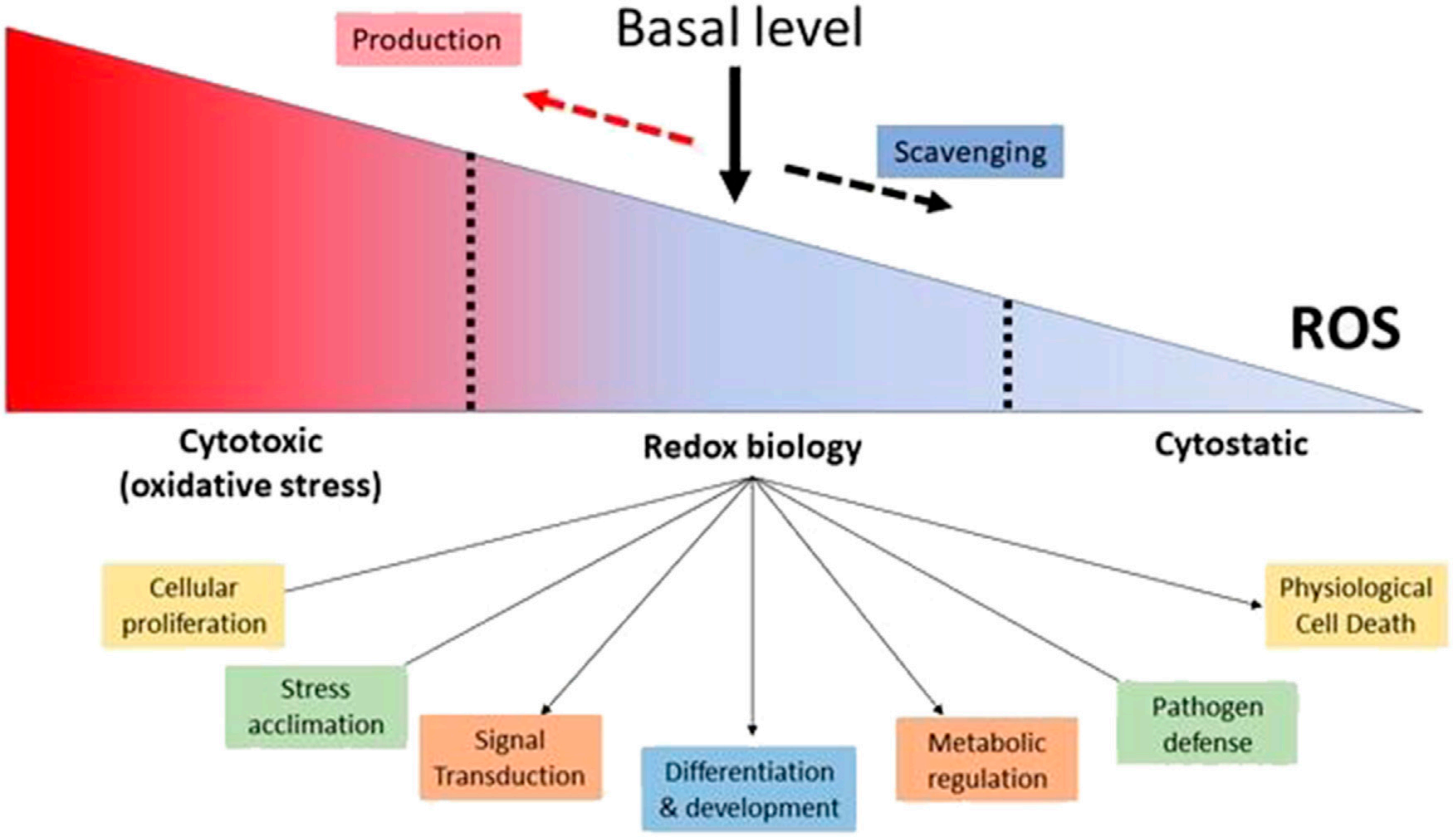
Maintaining a Basal Level of ROS in Cells is Essential for Proper Cellular Function. (Adaptation and modification from Mittler, 2017).

Furthermore, both intracellular and extracellular ROS agents have the ability to influence gene expression (Singh et al., 2019) In reaction to low quantities of H_2_O_2_, certain regulatory proteins, such as protein kinase B, can change the phosphorylation process. Plants respond to H_2_O_2_ by interacting with protein-DNA within the promoter region of antioxidant genes. The antioxidant-responsive element (ARE; TGACTCA), nuclear factor kappa B (NF-B), and abscisic acid-responsive element 2 (ACGT) are among these genes. (Checa and Aran, 2020). Other activities of ROS in plants include direct pathogen killing, cell wall construction, and programmed cell death activation, in addition to promoting the activation of defense genes (Mhamdi and Van Breusegem, 2018) ROS have been shown to inhibit cell division in yeast and mammalian cells, and ROS has been shown to inhibit cell cycle progression (Qi and Zhang, 2020) The revelation that O2- plays an important role in the battle against invading germs, effectively serving as a broad-spectrum antibiotic, offers as a crystal-clear and forceful demonstration of how ROS may be used for good (Bardaweel et al., 2018) Plants respond to pathogen invasion with a number of defensive mechanisms, including a “oxidative burst” that creates massive amounts of ROS quickly and transiently (Kapoor et al., 2019) As a result, ROS plays several, and often contradictory, functions throughout diverse cellular processes. The combination between ROS-producing mechanisms and the complex regulatory actions of cellular antioxidants produces the steady-state amount of ROS within cells, which is critical. It has been demonstrated that ROS and redox signaling regulate cellular functions and occur within a certain range of ROS concentrations. ROS levels that are too high are cytotoxic, whereas ROS levels that are too low are considered to be cytostatic. Therefore, appropriate ROS and redox signaling in cells requires a baseline level of ROS, which is maintained by the equilibrium between ROS generation and ROS scavenging. (Mittler, 2017) (Figure 2).

## 3 Oxidative DNA damage and epigenetic mark

Eukaryotic cells have developed incredibly complex methods to promptly adjust the expression of their genes in response to environmental changes. A wide variety of cellular stress serves as fundamental signals transduced into the cytoplasm, eventually affecting the expression of specific genes in the cell nucleus. The generation of ROS, also known as oxygen free radicals, is one such manifestation of cellular stress. Due to their continual modification of DNA’s base composition, ROS constantly threaten the structure, function and stability of the genetic material (Poetsch, 2020) these damages not only impair genome function but may also act as an epigenetic mark to help regulate gene expression (Gruber et al., 2018) The deterioration of pathophysiological conditions can be exacerbated when there is an imbalance in oxidative DNA damage and the resulting mutagenesis processes. These processes are closely linked to aging, inflammation, and the progression of various age-related ailments such as cancer and neurological disorders (Nissanka and Moraes, 2018; Barnes et al., 2019) This section will briefly discuss several aspects of epigenetic marks that are contributed by oxidative damages caused by ROS.

### 3.1 Influence on DNA transcription

The oxidation of DNA and associated proteins caused by ROS activity has long been recognized to affect DNA transcription. Nuclear factor erythroid 2-related factor 2 (NFE2L2 or NRF2), a basic leucine zipper transcription factor that binds antioxidant response elements (AREs) present in the promoter of several antioxidant enzymes, is a crucial regulator of the oxidative stress response. By doing so, NRF2 activates the transcription of the AREs-containing genes (Hine and Mitchell, 2012). In the body, NRF2 is coupled with its repressor Kelch-like ECH-associated protein 1 (KEAP1), which is then linked to CULLIN 3 (CUL3) and RING-box protein 1 (RBX1)/E3-ubiquitin ligase to create the KEAP1/CUL3/RBX1 E3-ubiquitin ligase complex, which promotes the destruction of NRF2 by the proteasome (Baird and Yamamoto, 2020; Szczesny-Malysiak et al., 2020). When exposed to oxidizing stimuli, KEAP1’s cysteine residues are bound by ROS, changing the protein’s structure. As a result, NRF2’s ubiquitination is inhibited, it is translocated to the nucleus, and it binds to ARE areas in the promoter region of antioxidant genes, triggering the transcription of those genes (Hine and Mitchell, 2012; Baird and Yamamoto, 2020). Due to its ability to impede apoptosis and hence stimulate tumor cell proliferation, invasion, and chemoresistance, the NRF2/KEAP1 pathway is also essential in the development of cancer. As a result, several cancer types have the NRF2/KEAP1 pathway as a primary target for chemotherapy (Zimta et al., 2019; Tossetta et al., 2022). In fact, studies have shown that higher levels of NAD(P)H quinone dehydrogenase 1 (NQO1) and NRF2 expression are associated with higher-grade cancers such as endometrial and cervical carcinomas as opposed to healthy tissue and precancerous lesions (Osman et al., 2015). NQO1 and NRF2 may be valuable biomarkers for early diagnosis, prognostic, and therapeutic strategies in patients with cervical and endometrial carcinomas, in line with additional research showing that NRF2 overexpression was significantly associated with high-grade and advanced-stage endometrial and ovarian carcinomas (Cho et al., 2017; Tossetta et al., 2022; Tossetta and Marzioni, 2023). Additionally, an increase in ROS leads to a variety of reactions in various signaling pathways, such as MAPK (Mitogen-activated protein kinase), NF-κB (nuclear factor kappa-light-chain-enhancer of activated B cells), STAT3 (Signal transducer and activator of transcription 3), or PPAR (Peroxisome proliferator-activated receptor gamma). In response to oxidative stress, these signaling pathways encourage the expression, proliferating, and survival of antioxidant genes, which facilitate the growth of cancer cells. Although each of these signaling pathways can independently control the expression of antioxidant genes, they are also all interconnected, demonstrating the intricate regulatory system that governs the response to oxidative stress.

### 3.2 Influence on DNA methylation

DNA methyltransferases (DNMTs), an epigenetic regulator, are primarily responsible for alterations in DNA methylation resulting from oxidative stress. According to several studies, hydrogen peroxide-mediated oxidative stress increases the activity of DNMT1 and its ability to bind to the promoters of tumor suppressor genes as Runx family transcription factor 3 (RUNX3) (Nishida and Kudo, 2013). Due to the interference with DNMTs’ capacity to bind DNA, hydroxyl radicals (HO•) also encourage global hypomethylation. (Kreuz and Fischle, 2016). As oxidative stress levels rise, iron’s catalytic cycle is altered, inhibiting DNA demethylases of the Ten Eleven Translocation (TET) family and raising DNA methylation levels (Franco et al., 2008). In addition, 8-hydroxydeoxyguanosine (8-OHdG) levels are elevated in some cancer types under conditions of high oxidative stress brought on by ROS production. The chromatin active state transforms into the chromatin repressive state when 8-OHdG induces a conformational change. Consequently, due to alterations in the methylation patterns of tumor suppressor genes, 8-OHdG might promote carcinogenesis (Nishida et al., 2013). Moreover, 8-OHdG prevents DNMTs from binding to DNA, which causes the entire genome to be hypomethylated (Ziech et al., 2011; Udomsinprasert et al., 2016).

Additionally, earlier research showed how epigenetic regulation of ROS and its connection to Alzheimer’s disease were related. Sung et al. and Chouliaras et al. have demonstrated that APP-related mutations produce an epigenetic shift in an AD model cell line in addition to a general decrease in DNA methylation in the hippocampus of *postmortem* AD patients (Sung et al., 2011; Chouliaras et al., 2013). Gu et al. investigated the potential causes of this epigenetic change in AD patients (Gu et al., 2013). Hydrogen peroxide treatment resulted in a considerable rise in histone acetylation and a decline in DNA methylation in neuroblastoma cells. Increased amyloid beta precursor protein (APP) and beta-secretase 1 (BACE1) transcription was the result of this histone hyperacetylation and DNA hypomethylation, potentially as a result of an increase in NF-B activity (Gu et al., 2013). This study demonstrates how epigenetic mechanisms used by oxidative stress can result in the creation of amyloid beta (Aß) plaques and suggests promising therapeutic avenues for this pathway. Furthermore, tobacco smoke is one factor that significantly contributes to oxidative stress and, consequently, to changes in the epigenetic machinery involving methylation (Mecocci and Polidori, 2012). In addition to being a powerful carcinogen, cigarette smoke produces large amounts of ROS, which promote the growth of illnesses such as chronic obstructive pulmonary disease (COPD), which also changes DNA methylation patterns (Jeon et al., 2011; Mecocci and Polidori, 2012).

### 3.3 Influence on the regulation of ROS-producing enzyme

Oxidative DNA damage results from the creation of particular ROS by the enzymatic activities of the epigenetic circuitry. Lysine-specific demethylase 1 (LSD1) is one of these enzymes that removes methyl groups from histones. Surprisingly, LSD1 creates ROS as a byproduct of its enzymatic action (Perillo et al., 2020) Such oxidative stress occurs quite close to the DNA. Estrogen-induced gene expression is one example of how this pathway influences gene regulation (Hao et al., 2018) The essential mechanism relies on the catalytic function of 8-oxoguanine DNA glycosylase-1 (OGG1) and the structural modifications induced by topoisomerase IIb on the promoter. The extensive enhancer and promoter capabilities of LSD1 indicate that similar methods could be valuable for investigating a diverse range of genes. Another illustration is the dual oxidase 1 (DUOX1) enzyme, which contributes significantly to the generation of ROS in the airways and is frequently suppressed in human lung cancer (Ling et al., 2014). Additionally, it was demonstrated that DUOX1 expression was lower in HCC than in healthy reference tissues, and this lower expression was associated with CpG island methylation in the promoter of the gene. Following 5-Aza-dC therapy, the expression of DUOX1 was restored. This reduced the proliferation and colony formation of cancer cells brought on by an increase in ROS levels and a cell cycle arrest in G2/M (Ling et al., 2014).

As proven by the aforementioned mechanistic evidence, oxidative DNA damage functions as an epigenetic marker in gene regulation and can interact with other epigenetic processes. Because mutations that arise during development are passed down to successive generations of cells, the epigenetic impact of oxidative DNA damage during differentiation may be especially severe (Schumacher et al., 2021).

## 4 Oxidative stress and pathophysiology

### 4.1 Metabolic diseases

#### 4.1.1 Atherosclerosis

According to the existing research, oxidative stress and atherogenic dyslipidemia are important variables in the evolution of atherosclerosis and its negative consequences, such as cardiovascular disease (CVD). Atherosclerosis is a chronic condition characterized by the buildup of lipids in the arteries, resulting in plaque development (Libby, 2021) Hypercholesterolemia, or excessive cholesterol levels in the blood, alters the permeability of the artery endothelium. This increases the likelihood of lipids, particularly low-density lipoprotein cholesterol (LDL-C), entering the artery wall (Vekic et al., 2022) Monocytes that migrate into the subendothelial space via diapedesis acquire macrophage properties, resulting in foamy macrophages. Monocyte-derived macrophages have the ability to oxidize low-density lipoprotein (LDL) in the subendothelial region, which is harmful to arterial cell cells (Prasad and Mishra, 2022) Macrophages will consume oxidized LDL via scavenger receptors, resulting in the formation of lipid-laden foam cells (Singh et al., 2022).

While the specific origins of atherosclerosis are unknown, most ideas about its onset and development concentrate around interrupting normal regulatory systems, resulting in oxidative stress. Numerous studies have indicated that oxidative stress is important in the development of atherosclerosis (Marchio et al., 2019; Poznyak et al., 2020a) The phrase “redox status” refers to an imbalance between antioxidant-capable species such as ROS, nitrogen (RNS), halogen species, and non-radical and free radical species (Sies et al., 2022) These imbalances can directly damage cellular proteins, lipids, and DNA or activate signaling pathways that promote cell death (Brillo et al., 2021) The walls of blood arteries possess various systems that generate ROS. Various systems within the walls of blood arteries are responsible for generating ROS, including XO, eNOS, enzymes in the mitochondrial respiratory chain, and NADPH oxidase (NOXs) (Daiber et al., 2021; Burtscher et al., 2022; Leyane et al., 2022) These oxidases are complex enzymes composed of multiple subunits, which produce superoxide from molecular oxygen by utilizing NADPH as an electron supplier. They comprise two subunits associated with the membrane, along with several modulatory subunits located in the cytoplasm (Saxena and Jha, 2021; Sharma et al., 2022) Contrary to NOX1 and NOX2, NOX4 only requires p22phox (the ubiquitous protein encoded by the CYBA gene located on the long arm of chromosome 16 at position 24) and releases hydrogen peroxide instead of superoxide (Moghadam et al., 2021) Three NOX isotypes are expressed in the vascular smooth muscle cells (VSMC) that line the blood vessels of mice; two of these isotypes, NOX2 and NOX4, are preferentially expressed in endotheliocytes (Thiriet, 2015; Thiriet, 2018).

According to a recent study, NOX enzymes perform various functions in atherogenesis (Poznyak et al., 2020b) XO utilizes the molecule oxygen as an electron acceptor to generate hydrogen peroxide and superoxide (Bortolotti et al., 2021; Letourneau et al., 2021) Endothelial XO expression is increased in response to proatherosclerotic stimuli such as angiotensin II (Ang II) treatment and oscillatory shear stress (Bartekova et al., 2019; Polito et al., 2021). SOD2 and GPx1 are manganese-dependent superoxide dismutases that convert typical levels of superoxide generated by mitochondrial oxidative phosphorylation into hydrogen peroxide and water, respectively (Palma et al., 2020; Napolitano et al., 2021). Additionally, human atherosclerosis has been linked to mitochondrial oxidative stress (Petrucci et al., 2022) Mitochondrial ROS are utilized extensively as cellular messengers in signaling cascades. Therefore, mtROS are crucial for controlling cellular processes such as signal transmission, gene expression, and stress responses (Shadel and Horvath, 2015; Kasai et al., 2020) Hydrogen peroxide and superoxide are the two main types of mtROS. In oxidative phosphorylation, molecular oxygen can receive electrons from complex IV that are moving down the electron transport chain (ETC) and are then transferred to produce water. Nevertheless, mitochondrial proteins that are found early in the, ETC., cause electrons to “spill” onto oxygen, which results in the formation of mtROS. Although Complex I is thought to be the primary location of mtROS production, they can also come from Complexes II or III (Quinlan et al., 2013) Both forward and reverse electron transfers (FET and RET) can cause ROS to be produced by Complex I (Scialò et al., 2017) Ubiquinone (CoQ) obtains electrons from a variety of sources, such as complexes I and II, during FET. However, mtROS can be produced by electron leakage during NADH oxidation at Complex I’s flavin mononucleotide (FMN) site (Jastroch et al., 2010) Under resting conditions, mtROS produced during FET function as redox messengers. ROS can also develop at the CoQ binding site in CI. However, when the proton motive force (*Δ*p) across the mitochondrial inner membrane is elevated and the pool of ubiquinone electron carriers is severely depleted, RET takes place in Complex I. By reducing NAD + to NADH, ubiquinol (reduced CoQ) provides electrons to complex I. Superoxide is created when some of these leaking electrons reduce oxygen. When there is reduced oxidative phosphorylation capacity due to cellular stress or mitochondrial injury, RET may take place.

#### 4.1.2 Dyslipidemia

Dyslipidemia is a condition with an increased level of total or LDL cholesterol or a reduction in high-density lipoprotein (HDL) cholesterol level, leading to an imbalance of lipids (Pappan and Rehman, 2021) Increased endothelial ROS production is intimately linked to atherogenic dyslipidemia. Smaller LDL particles have been associated with a more significant state of oxidative stress, according to studies, which may be present in asymptomatic people (Kovačević et al., 2022) In addition, smaller LDL particles were linked to circulating inflammatory biomarkers before any clinical proof of atherosclerosis (Antoniazi et al., 2022). These results support the idea that lipid peroxidation and inflammation play a highly cooperative role in the initiation and development of atherosclerosis (Viktorinova et al., 2021). *In vitro* assays have demonstrated direct proof that smaller, denser LDL particles are more susceptible to oxidation (de Queiroz Mello et al., 2018; Blagojevic et al., 2022) *In vivo*, LDL oxidation’s mechanisms have also been extensively studied (Hernáez et al., 2019; Wang et al., 2020; Itabe et al., 2021).

While oxidatively modified LDL can contribute to increased oxidative stress, it also possesses the ability to promote inflammation, blood clot formation, and programmed cell death (Jaisha et al., 2021) Numerous studies have established a significant association between atherosclerosis and CVD and elevated levels of oxidized LDL in the bloodstream, autoantibodies targeting oxidized LDL, or LDL particles modified by malondialdehyde (MDA) (Dunér et al., 2021; Porsch et al., 2021; Berger and Naseem, 2022) According to a recent study by Yamashita et al., higher oxidized LDL particles are linked to thrombosis and plaque instability (Yamashita et al., 2010) High levels of oxidized LDL in the blood have also been found to be independent predictors of subclinical and clinical atherosclerosis in research on clinically healthy individuals (Kigka et al., 2021; Sweeney et al., 2021) As a result, exceeding LDL cholesterol levels, oxidized LDL levels have been postulated as a biomarker of cardiovascular disease risk. In another research, Ishigaki et al. discovered that removing oxidized LDL from mice’s blood completely stops the progression of atherosclerotic disease (Ishigaki et al., 2009) Their research may open new treatment avenues for lowering the prevalence of atherosclerotic illnesses.

### 4.2 Neurodegenerative diseases

#### 4.2.1 Parkinson’s disease (PD)

Parkinson’s disease is a chronic neurodegenerative disorder that usually affects the geriatric population that is 60 years old and above (Aarsland et al., 2021) It usually presents with bradykinesia or slow movement and other symptoms of resting tremor or rigidity (Heijmans et al., 2019) There is yet little knowledge of the elements that might contribute to oxidative stress in PD. However, increased ROS production in PD is closely linked to mitochondrial dysfunction (Blesa et al., 2015; Weng et al., 2018) The discovery that mutations in the genes encoding proteins like -syn, parkin, DJ-1, or PINK are associated with familial types of PD raises the question of how oxidative stress and dopamine (DA) cell damage are related to mitochondrial dysfunction (Gonchar et al., 2019; Ejma et al., 2020) The convergence of these proteins on mitochondrial dynamics reveals a shared role in the mitochondrial stress response, which may serve as a potential physiological underpinning for the pathology of PD. These findings indicate that mutations in these genes impact the functionality and stability of the mitochondria and are linked to greater levels of oxidative stress (Quinn et al., 2020; Dorszewska et al., 2021) Oxidative stress impacts the activity of mitochondria, lysosomes, and proteasomes, which dictate cellular responses to oxidative damage. Consequently, this can lead to incorrect protein folding. This misfolding might hinder the proper unfolding and removal of some proteins by systems responsible for protein degradation, like the ubiquitin-proteasome system or autophagy (Choi and Gandhi, 2018; Picca et al., 2021) Protein misfolding may be a significant factor in the emergence of harmful events linked to the neurodegenerative process of PD, together with the failure of these protein degradation mechanisms.

Moreover, microglia, the predominant immune cells found in the brain, along with astrocytes and oligodendrocytes, albeit to a lesser degree, are linked to the degeneration of neurons in PD (Badanjak et al., 2021; Leyane et al., 2022) Specific endogenous proteins and environmental contaminants can induce microglia to become too active and generate ROS, which can be neurotoxic (Afsheen et al., 2022) A growing body of evidence suggests that activation of various enzymes, such as NOX2 in microglia, is neurotoxic not only by producing extracellular ROS that harm nearby neurons but also by commencing redox signaling in microglia that intensifies the pro-inflammatory response (Pajares et al., 2020; Begum et al., 2022) Therefore, reducing the harmful effects of oxidative stress in neurodegenerative disorders relies on discovering prospective therapeutics that can postpone the neurodegenerative process. In the coming years, neuroprotective interventions must focus on various pathogenic mechanisms, including neuroinflammation and mitochondrial dysfunction.

#### 4.2.2 Alzheimer’s disease (AD)

The hallmark of AD is a progressive decline in cognitive abilities associated with a considerable decrease in brain volume in AD patients compared to healthy controls (Manap et al., 2019) An important aspect of AD and a link to oxidative stress is oxidative damage to neuronal lipids and proteins. The brain’s susceptibility to oxidative stress is thought to be a major contributing factor to AD (Rummel and Butterfield, 2022) The Aß may bind to loosely bound metal ions (copper and ion), which are abundant in the brain and have a high concentration in the senile plaques of AD patients, stimulating the generation of ROS very effectively (Kim and Lee, 2021; Squitti et al., 2021) The fact that Aß promotes oxidative stress and accelerates the development of Aß further demonstrates the relationship between oxidative stress and Aß (Cheignon et al., 2018) The evidence favoring oxidative stress’s central role in AD is convincing. This evidence demonstrates that various clinical and pathological symptoms of AD, including the deposition of Aß plaques, the formation of neurofibrillary tangles, metabolic dysfunction, and cognitive decline, arise following a prolonged period of gradual accumulation of oxidative damage (Rao et al., 2020) This section will further discuss the two hypotheses on the association between Aß and oxidative stress.

#### 4.2.3 Oxidative stress enhances Aß production

**FIGURE 3:**
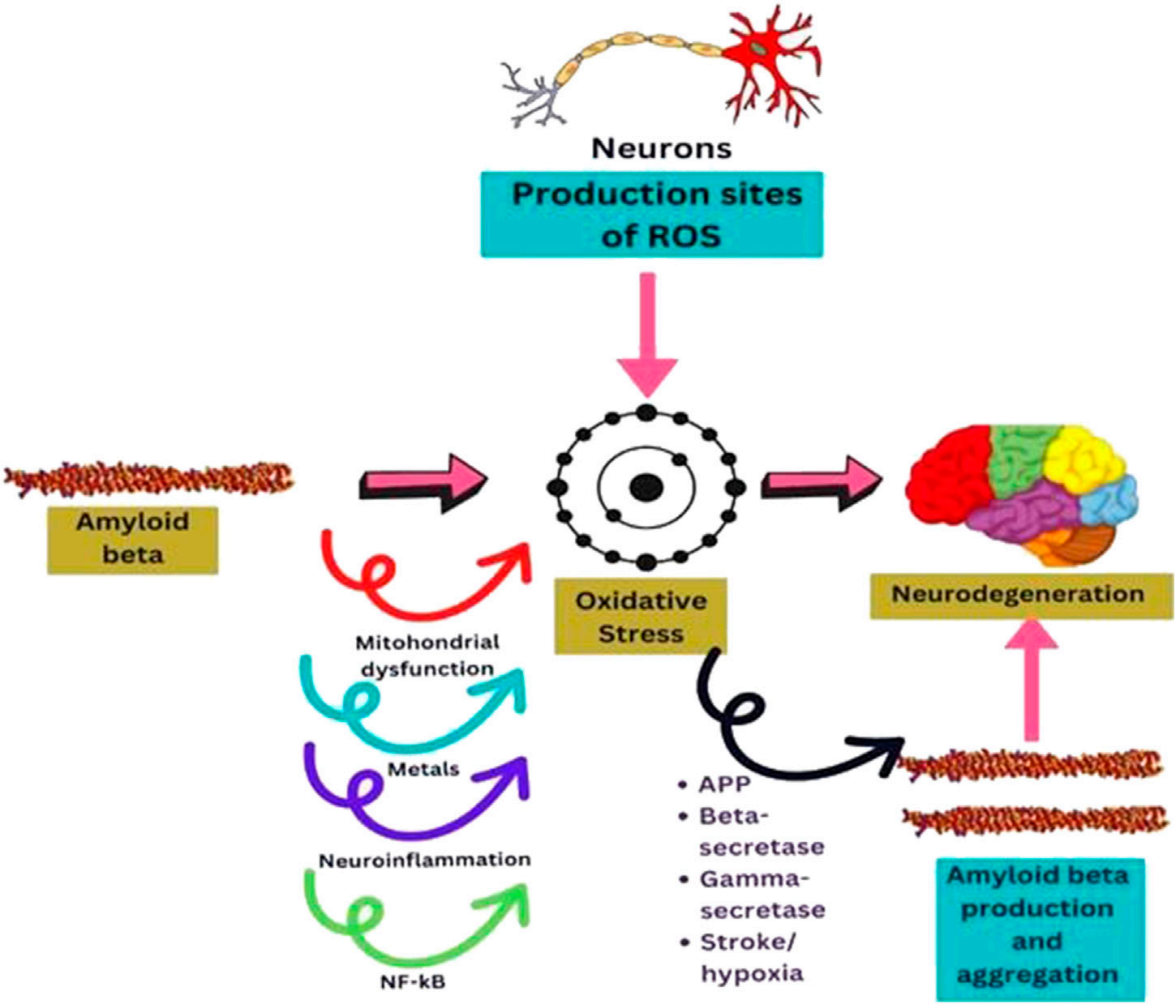
Diagram sketching mechanisms by which oxidative stress mediates AB production, or AB induces oxidative stress in neurodegeneration.

In contrast to other cells, neurons consume a lot of oxygen, which drives their mitochondria to generate significant ATP. This feature is tightly related to the fact that they are spots where ROS are produced and are especially vulnerable to the damage they cause (Bhatt et al., 2021). It is more likely that oxidative stress is a precursor to the development of AD pathology from normal aging due to the substantial oxidative damage seen in mild cognitive impairment (MCI) (Ahmed and Braidy, 2021). These hypotheses suggest that enhanced ROS production may play a crucial role in mediating synaptic loss and, in turn, causing senile plaques (Uddin et al., 2022) (Figure 3).

The idea that oxidative stress is a typical mechanism mediating the buildup and toxicity of Aß has been supported by a large body of literature (Bhatia and Sharma, 2021; Ionescu-Tucker and Cotman, 2021; Sharma and Kim, 2021). Oxidizing agents enhance APP expression (Rahman et al., 2020; Tamagno et al., 2021) and raise intracellular and secreted Aß levels (Promyo et al., 2020). Earlier, it was noted that oxidative stress promoted the activity and expression of BACE 1 (Tong et al., 2005). Additionally, there is a substantial relationship between BACE1 activity and oxidative stress markers in sporadic AD brain tissues (Tamagno et al., 2021), where BACE1 levels are significantly higher than in healthy brain tissue (Cheng et al., 2014; Taylor et al., 2022). It has been demonstrated that oxidative stress, which develops with aging, can stimulate both secretases’ activity (Beta and gamma secretases) and mediate Aß’s hyperproduction. By augmenting the activity of Presenilin 1 (PS1), the catalytic subunit of secretase, oxidative stress is the sole known factor that can increase the cut on APP controlled by Y-secretase. These findings show the existence of a feedback loop in which elevated Y-secretase activity leads to elevated BACE 1 expression (Price and Borchelt, 2003).

On the other hand, it is well-known in the literature that those with cerebral infarction or strokes are more susceptible to contracting AD (Wang et al., 2021). According to a theory, hypoxia can influence how APP is metabolized by enhancing beta and gamma-secretase activity. The first evidence of a significant increase in BACE1 expression and activity due to hypoxia came from Sun et al. (Sun et al., 2006). Furthermore, hypoxia can boost the activity of Y-secretase. As a result, hypoxia-inducible factor (HIF)-1 binds to the promoter of anterior pharynx-defective phenotype (APH-1), a crucial part of the secretase Y complex, and causes hyperregulation of this component (Hassan and Chen, 2021). Together, these findings demonstrate that hypoxia increases both secretases’ activity, which leads to an excess of Aß synthesis and the development of plaques in both *in vivo* and *in vitro* models.

#### 4.2.4 Aß induces the development of oxidative stress

Aß mediates oxidative damage through a variety of pathways that have been documented in the literature (Figure 3). Aß disrupts healthy mitochondrial function, leading to malfunctioning and oxidative stress that could affect neuronal function (Guo et al., 2013). Neurons are energy-intensive cells that execute a variety wide range of tasks, including the production of action potentials, nerve signaling, and axonal transport (Schwarz, 2013). The peptide Aß induces ROS production at the mitochondrial level and hinders ROS clearance. It has been demonstrated that Aß can impede mitochondrial superoxide dismutase (MnSOD), primarily responsible for detoxifying anion superoxide and preventing oxidative damage (Flynn and Melov, 2013; Sharma and Kim, 2021). Additionally, several scientists showed that mitochondrial DNA is altered in aged and AD patients (Klein et al., 2021) due to the direct implication of Aß on mitochondria. Oxidative stress is linked to mitochondrial DNA (mtDNA) damage, and it was found that the genes in mitochondrial complex I of oxidative phosphorylation are downregulated in the brain tissue of AD patients (Marmolejo-Garza et al., 2022).

Besides, the homeostasis of metals, including iron (Fe), copper (Cu), and zinc (Zn), is another process known to be dysregulated in AD (Das et al., 2021). The blood-brain barrier strictly controls the concentration of these metals, but in AD patients, their levels considerably rose (Das et al., 2021; Lippi et al., 2021). There are traces of these metals found in Aß plaques (Patel and Aschner, 2021), and Aß can reduce Fe (III) or Cu (II) to promote the generation of H_2_O_2_, which tends to trigger oxidative stress events in AD (Bush and Tanzi, 2008). Moreover, zinc is also prevalent in amyloid plaques, indicating its involvement in AD. *Ex vivo* research revealed that Zn (Aß) complex oligomers prevented hippocampus long-term potentiation (LTP) in a transgenic mouse strain via generating ROS (Ma et al., 2011). Heme is yet another significant iron-containing component linked to the etiology of AD. According to a previous report, compared to healthy aging controls, AD patients showed lower hemoglobin levels and smaller cell volumes (Altinoz et al., 2019). Heme can bind to Aß, and this complex has been shown to have peroxidase activity that can damage serotonin and DOPA, establishing an intriguing connection between heme and oxidative stress in AD (Atamna and Frey, 2004). This discovery implied that the iron buildup seen in AD might be strictly related to heme insufficiency (Hadzhieva et al., 2014; Gozzelino, 2016).

### 4.3 Cancer

#### 4.3.1 Oxidative stress increases cancer risk

Numerous research has indicated that tumors produce a higher amount of ROS compared to healthy cells. This phenomenon aids in the development of tumors by enhancing the survival and multiplication of tumor cells and causing DNA damage and genetic instability. It is believed that the interplay between signaling mediated by tumor necrosis factor-alpha (TNF-α) and ROS generated by myeloid cells plays a role in chronic inflammation, potentially promoting the onset of cancer (Canli et al., 2017). It is also widely established that DNA can be altered by prolonged exposure to high ROS levels and that oxidative DNA damage has a distinct “catalog of somatic mutations in cancer” (COSMIC) mutation signature (Rose Li et al., 2020). The most compelling proof that ROS could increase the likelihood of cancer arises from removing O2-scavenging enzymes. This suggests insufficient antioxidant defenses to counter oxidative stress may lead to elevated cancer risk. Consequently, mice lacking both copies of the cytoplasmic superoxide dismutase 1 (SOD1) gene or having one copy deleted for the mitochondrial superoxide dismutase 2 (SOD2) gene exhibit noticeable oxidative harm and experience spontaneous cancer development (Gill et al., 2016).

Regarding the enzymes that eliminate H_2_O_2_, the absence of specific genes responsible for peroxiredoxin (Prdx) and selenium-dependent Gpx enzymes enhances the vulnerability to cancer. A previous study revealed that mice lacking one or both copies of the Prdx1 gene exhibited higher levels of oxidative DNA damage. Additionally, mutant mice that aged demonstrated a higher incidence of various cancers, including hepatocellular carcinoma, fibrosarcoma, osteosarcoma, islet cell adenoma, lung adenocarcinoma, and breast adenocarcinoma, compared to Prdx1^+/+^ mice of the same age (Rani et al., 2012). Contrarily, PRDX2 probe 39,729_at, PRDX4 probe 201,923_at, and PRDX6 probe 200,845_s_at, mice do not spontaneously acquire cancer (Hampton et al., 2018). In contrast, Gpx1/2 knockout animals are generally healthy in the lab despite being predisposed to ileocolitis during weaning and developing commensal microbiota-dependent ileal and colonic tumors by the age of 6 months (Chu et al., 2004; Chu et al., 2020).

Additionally, many carcinomas have brought attention to the crucial role of dietary heme contributes to cancer. Most of the dietary heme is absorbed in the upper small intestine. Heme is converted into biliverdin, carbon monoxide (CO), and iron (Fe2+) after absorption by the enzymes heme oxygenases (HMOXs), which are then scavenged by the protein ferritin (Sjoblom et al., 2006). Cells build up free heme and labile iron in the presence of high free heme levels, which causes both ferritin and HMOXs to become saturated and cause a range of cytotoxic effects on the intestinal mucosa (BS et al., 1991). For instance, heme can cause surface epithelial cells to remain susceptible to cytotoxic damage that alters surface-to-crypt signaling, leading to hyperproliferation and ultimately hyperplasia of crypt cells in mice supplied heme (Brouwer et al., 2004). Moreover, the buildup of free heme and labile iron triggers the pathological oxidation of proteins, lipids, and DNA by ROS. It has been established that proteins involved in the formation of colorectal cancer (CRC) are redox-sensitive and that ROS-induced DNA damage and gene alterations cause CRC (Wolff and Nourooz-Zadeh, 1996). Chronic inflammation can result in significant increases in peroxynitrite (ONOO−) levels, which, when combined with CO_2_, create nitrosoperoxycarbonate, which then breaks down into CO_3_ d and dNO_2_ and starts to oxidize selectively and nitrate guanine in DNA, causing guanine thymidine crosslinks (Shafirovich and Geacintov, 2017). Given that an increase in the baseline levels of ROS is linked to a higher likelihood of developing cancer, it can be inferred that a continuous decline in antioxidant capacity is similarly associated with comparable risk. As the removal of glutamate cysteine ligase (GCLC) or glutathione synthetase (GSS) is fatal during embryonic development, establishing the role of (GSH) in modifying the risk of carcinogenesis becomes challenging. In mutant mice, the levels of GSH are only 15% of those observed in wild-type mice, indicating a less pronounced impact of the knockout of the Glutamate-Cysteine Ligase Modifier Subunit (GCLM(−/−)) on GSH levels in these tissues (Killoy et al., 2018; Chen et al., 2022). Although.

GCLM (−/−)/hSOD1^WT^ mice do not spontaneously form tumors, their prepared fibroblasts exhibit elevated levels of ROS and DNA damage and upregulated levels of Tp53 and p21 (Hayes et al., 2020).

### 4.4 Respiratory syndrome

#### 4.4.1 Oxidative stress in the pathogenesis of COVID-19

The SARS-CoV-2 infection pathogenesis is still shrouded in many unknowns and uncertainties, and the patient’s unique immunological response to infection remains the central mystery. Since oxidative stress is one of the mechanisms used to explain the cell and tissue damage caused by influenza virus, it might influence how the outcome is determined. Given that COVID-19 infection can cause oxidative damage in various organs and tissues, SARS-CoV-2 infection can cross the cell membrane via the angiotensin 1 converting enzyme 2 (ACE2) receptor. This protein is extensively represented in numerous cells and tissues in the body (Beye et al., 2021). Previous studies showed that the damaged air spaces, pneumocytes, and alveolar macrophages in SARS-CoV-infected human lung tissues explicitly produced oxidized phospholipids and generated ROS (Gediz Erturk et al., 2021). Furthermore, it has been demonstrated that TLR4-TRIF-TRAF6 expression and cytokine production is triggered by the oxidized phospholipids in macrophages, which modulate the severity of lung injury (Sorokin et al., 2020). According to a previous report, the SARS-CoV-1 protease-3CLpro induces the ROS-activated NF-κB signal transduction pathway, which may play a role in the progression of the SARS-CoV infection (Wieczfinska et al., 2022).

The multifunctional ACE2 is also referred to as the SARS-CoV-2 cell entrance receptor. In addition to serving as the cellular receptor for SARS-CoV-2 spike proteins, ACE2 has a dual-edged function in the SARS-CoV-2 infection because it protects tissues from COVID-19’s oxidative and inflammatory damage (White, 2021). This enzyme suppresses the NADPH oxidase stimulant angiotensin II. Angiotensin one to seven, a byproduct of ACE2 enzyme activity, also has a potent antioxidant effect (Rush and Aultman, 2008; Tamama, 2021). The virus enters the cell by attaching to the ACE2 receptor, and ACE2 expression is downregulated after virion-membrane fusion and attachment (Perrotta et al., 2020). In contrast to previous viral infections, the viral protein spike coupling with ACE2 results in increased angiotensin II (Ang II) synthesis and stimulation of NADPH oxidase, which in turn enhances oxidative stress mechanisms while also releasing inflammatory mediators (Banu et al., 2020). Through the high affinity and subsequent binding of the SARS-CoV-2 virus to ACE2, angiotensin II production is elevated upon SARS-CoV-2 infection (Verdecchia et al., 2020). It has been demonstrated that ACE2 in SARS-CoV-infected cells is also implicated in the modulation of post-infection processes like immune response, viral genome replication, and cytokine release (Astuti and Ysrafil, 2020). An earlier investigation revealed that overexpression of ACE2 inhibits the endothelium’s ability to produce ROS and Ang II-induced Nox2 expression (Zhang et al., 2018). In normal individuals, ACE2 regulates blood pressure, inflammation and promotes lung homeostasis by producing angiotensin 1–7 (Penninger et al., 2021). In chronic respiratory settings, ACE2 downregulation, however, might stop SARSCoV-2 host cell contact (Restini et al., 2021).

The main cytokines produced due to the immune response in severe COVID-19 are interleukin (IL)-1, IL-2, IL-6, and tumor necrosis factor (TNF) (Rokni et al., 2020). Additionally, interferon-gamma (IFN-ϒ) seems crucial for the antiviral response, even though the data indicate impaired interferon synthesis and release in severely ill SARS-CoV-2 patients. IL-1 is a well-known stimulator of the production of ROS and RNS (Seifried et al., 2007). Similarly, IL-2 promotes the production of nitrogen radicals (NO•) via RNS (Patel et al., 1999). Human neutrophils and monocytes are activated by IL-6, which increases the production of free oxygen radicals (Wu, 2020). IFN-ϒ and TNF act similarly to induce the production of RNS in humans (Seifried et al., 2007).

On the other hand, free oxygen radicals may enhance the synthesis of IL-6, and free nitrogen radicals (NO•) are at least partially accountable for their production (Seifried et al., 2007; Wu, 2020). It was also mentioned that patients with COVID-19 receiving intensive care unit treatment have higher fatality rates when their IL-6 levels are high (Gorham et al., 2020). The elderly population, whose antioxidative/nitrosative defense system is weakened by higher levels of ROS and nitrogen species, is especially vulnerable to the harmful effects of this virus. Moreover, individuals with severe COVID-19 infection can significantly benefit from treatments such as dexamethasone and tocilizumab, which specifically decrease the production of ROS and RNS (Albuquerque et al., 2022). Consequently, it is critical to thoroughly explore this aspect since it would be noteworthy to use antioxidants as prospective therapeutic agents.

## 5 Oxidative stress and animal models

Animals, especially mammals, have been shown to have remarkable similarities in anatomy and physiology with humans (Barré-Sinoussi et al., 2015). Due to this similarity, researchers can investigate possible mechanisms that could lead to problems and identify and assess therapies applied in animal models. This is an essential step before applying the treatments to humans. The investigation of all levels, from the molecular level to the physiological function in both conditions (diseased and healthy), is required to ensure that the mechanisms are fully understood. Some level of investigation used *in vitro* approaches to copy or mimic the tissue complex structures. However, some mechanisms require a whole organism to identify the function and systemic interaction between the organs. Animal models are the best accessible tool to examine the pathways that may contribute to that ailment or its therapy, as *in vitro* experiments cannot properly depict the interactions that may be involved (Singh and Seed, 2021). Identification of broad pathways or mechanisms is crucial to preventing adverse effects or, at the very least, minimizing them when treatments are given to patients. In this section, we’ll go over successful animal models that have been implemented in prior studies to demonstrate how ROS are generated as a result of potentially harmful agents like buthionine sulfoxamine-induced, carbon tetrachloride, and others. Future studies concerning how oxidative stress operates via these models and investigating potential therapies may leverage this knowledge. For example, 4-hydroxy-2-nonenal (4-HNE), a breakdown product of paraquat-induced oxidative stress in mice, could be employed as the biomarker of the lipid peroxidation pathway to adopt therapeutic approaches that link this route to oxidative stress.

### 5.1 Buthionine sulfoximine (BSO)

Glutathione is the most abundant non-protein thiol originating from the liver, a vital defense mechanism against oxidative stress. About 80%–85% of the cellular GSH is available in the cytosol, 10%–15% is present in the mitochondria, and a small percentage of GSH is present in the endoplasmic reticulum (Hwang et al., 1992). *γ*-glutamyltranspeptidase (GGT) is the only enzyme to hydrolyze the bond between glutamate and cysteine (Lu, 2013). Buthionine sulfoximine induced inhibits the *γ*-glutamylcysteine synthetase (γ-GCS), which causes a decreased intracellular concentration of GSH both *in-vivo* and *in-vitro* (Sajjadian et al., 2014). The Buthionine sulfoximine (BSO) irreversibly binds to *γ*-GCS, which is required during the condensation of glutamate and cysteine to produce GSH.

Vaziri et al.’s (2000) studid animals used male Sprague-Dawley rats with an average weight of 275 g for the trial. (Vaziri et al., 2000). The rats were randomly put in the oxidative stress or placebo-treated control groups. Based on the previous studies in the rats, BSO 30 mmol/L dose was used for the oxidative stress group. In contrast, the placebo-treated control group was supplemented with regular water, and administered via drinking water for 2 weeks (Meister, 1983). The primary purpose of this study was to increase the ROS by reducing the GSH level. The results from the rats treated with BSO expressed a nearly 3-fold reduction of total GSH content of the liver tissue and an increase in arterial blood pressure. The BSO-treated rats showed a sharp decrease in urinary nitrate plus nitrite (NOx) excretion, and after 7 weeks of BSO discontinuation, whereas, the NOx level returned to the baseline. Significant increases in nitrotyrosine in the plasma, liver, heart, kidney, and aorta were found in the BSO-treated rats. The reduction of NOx urinary excretion and the high concentration of nitrotyrosine in the rats’ plasma and organs exhibited that an interaction between nitric oxide (NO) and ROS could contribute to the pathogenesis of hypertension in BSO-treated rats (Eiserich et al., 1995).

In another study conducted by (Sajjadian et al., 2014) used BALB/c strain mice and divided them into three groups (Sajjadian et al., 2014). The untreated mice in the control group did not receive any chemicals. The BSO induced experimental group mice was administered intraperitoneal (IP) injection of 2 mmol/kg BSO for 35 days. The third group (Sham group) received 0.9% saline with a similar olume as the BSO induced experimental group mice. The main objective of this study was to identify the effect of BSO induced oxidative stress based on the histological structure of the testis, semen parameters, and testosterone secretion. High production of ROS caused damage to the cells, including spermatozoa (De Lamirande and Gagnon, 1995). The architecture of the spermatogenic epithelium of BSO induced experimental mice was disturbed, and degeneration of the vacuolar was seen in the spermatogenic epithelium. The control mice and sham groups were found to have normal structures of spermatogenic epithelium. The previous finding showed that the BSO administration affects the testes’ histological architecture and cellular damage (Pasqualotto et al., 2000). The BSO induced experimental mice showed significantly decreased GSH, CAT (catalase), SOD and GPx concentrations, while in the control and sham groups, all the values remained normal. GPx, a spermicidal antioxidant, could protect the sperm against peroxidative damage (Hsieh et al., 2006). However, BSO decreases the GPx concentration in the BSO treated experimental group, possibly leading to the infertility. Morphology and sperm count were reduced when the MDA level increased, which indicated that BSO inhibited the GSH, whereas increased ROS production (Lean et al., 2003).

Another animal model study conducted by (Reliene and Schiestl, 2006) used C57BL/6Jpun/pun mice for experiment (Reliene and Schiestl, 2006). The mice were separated into five groups: un-supplemented water (control group), 2 μm BSO, 20 μm BSO, 2 μm BSO +20 μm NAC and 20 μm NAC (N -acetyl- L–cysteine) for 18 days. The 2 μm and 20 μm BSO doses were chosen as the administration of BSO 2–6 mM/kg/day, in the pregnant rats via drinking water, were able to reduce the GSH without inducing any teratogenic effects towards the offspring (Reyes et al., 1995). The primary objective of this experiment was to establish the role of BSO-induced oxidative stress in carcinogenesis, leading to significant genomic rearrangements while reducing GSH levels without causing teratogenic effects. In the group treated with BSO doses (2 and 20 μm), the GSH concentration in mouse fetuses decreased compared to the untreated BSO group. Decreased GSH levels could amplify oxidative stress and affect other antioxidants like cysteine, which also exhibited reduced concentration in BSO-treated mice. The administration of BSO, resulting in oxidative stress, led to decreased levels of major thiol antioxidants, GSH and cysteine and increased the occurrence of 70 kb DNA deletions. The depletion of thiol antioxidants *in vivo* triggers permanent genetic alterations that can predispose individuals to cancer or other genetic disorders. Both animal models employed a shared pathway, inhibiting GSH to enhance ROS production. The advantage of utilizing BSO in animal models lies in its ability to target specific antioxidants while causing limited effects on other cellular components. However, BSO may not increase oxidative stress overall if antioxidants such as CAT, SOD, and GPx can compensate for GSH inhibition. A summary of the BSO experiment is presented in Table 2.

**TABLE 2:** Buthionine sulfoximine (BSO) induced oxidative stress animal model.

Animal model	Experimental animal	Duration	Dose/Amount	Targeted cell/organ	Outcome	Conclusion	References
Buthionine sulfoximine (BSO)–induced	Sprague–Dawley rat	2 weeks	30 mmol/L of BSO	Plasma, liver, heart, kidney and aorta	There is a significant reduction of GSH concentration in the animal model	The administration of BSO can induce oxidative stress by reducing the GSH concentration	Vaziri et al. (2000)
	BALB/c mice	35 days	2 mmol/kg of BSO and 0.9% of saline	Spermatogenic epithelium, testis	Administration of BSO reduced the GSH, CAT, GPx and SOD concentration	BSO increased oxidative stress, which causes damage to the spermatozoa cell	Sajjadian et al. (2014)
	C57BL/6J*p* ^un^/*p* ^un^ mice	18 days	2 μm of BSO, 20 μm of BSO, 2 μm of BSO +20 μm of NAC	DNA cells	Significant reduced of GSH concentration in the mice	Reduction of GSH synthesis increases oxidative stress which causes teratogenesis	(Reliene and Schiestl, 2006)

Abbreviations: GSH, glutathione; CAT, catalase; SOD, superoxide dismutase; GPx, Glutathione peroxidase; DNA, deoxyribonucleic acid.

### 5.2 Carbon tetrachloride (CCl4)

A hepatotoxic agent, carbon tetrachloride (CCl4) is metabolized by cytochrome p450 enzymes in the endoplasmic reticulum to produce metabolite, which is a highly reactive trichloromethyl radical (CCl3•). A highly reactive trichloromethyl peroxyl radical (•CCl₃O2) is formed when CCl3 rapidly reacts with oxygen (Ritesh et al., 2015). The CCl3• react with lipids to form lipid peroxidation products (Basu, 2003). Mitochondria and polyunsaturated fatty acids (PUFA) of the endoplasmic reticulum have a higher susceptibility to being oxidized by the free radicals, which affect the permeabilities of the plasma membrane, endoplasmic reticulum as well the mitochondrial, thus leading to cell damage (Weber et al., 2003). The cell damage could induce an inflammatory response which activates the neutrophil to increase the production of free radicals, thus increased the oxidative stress level and liver damage (Rizwan et al., 2014).

The animal models used to determine the CCl4-induced acute liver injury with the involvement of H_2_O_2_ were acatalasemic and control mice provided initially by previous researchers (Feinstein et al., 1968). Feinstein et al. (1968) study showed that the CAT activity of acatalasemic mice was less as compared to control mice; however, there was no difference in GPx in both groups. In another study, a single dose of 0.0125 mL of CCl4 was given via IP injection to acatalasemic and control mice (Wang et al., 1996). The acatalasemic mice showed a significant increase in aspartate transaminase (AST) and alanine transaminase (ALT) compared to the control mice after 18 and 24 h of CCl4 treatment. High AST and ALT activities may indicate CCl4-induced liver damage in both animals but with more intensity in acatalasemic mice. The CAT activity in the liver in CCl4-treated acatalasemic mice was lower as conmpared to CCl4-treated Wistar mice. This increases the cellular or tissue levels of H2O2, which possibly leads to the liver injury.

The animal model conducted by (Ritesh et al., 2015) used CCl4 to induce oxidative stress to determine its effect on the brain (Ritesh et al., 2015). Wistar rats weighed between 200–230 g were separated into two groups which were the experimental group and the control group. In the experimental group, the single dose, in which hepatotoxicity occurred, 1 mL/kg body weight of CCl4 was dissolved in sunflower oil (Srivastava and Shivanandappa, 2006). For the control group, the administration consisted of only sunflower oil. Both the control group and experimental group animals were orally gavaged for the traeatment. The lipid peroxidation showed a significant increase in the experimental rat’s brain regions to a maximum level of 159% in the forebrain and to the least level attained was at 62% in the midbrain, while the lipid peroxidation toward the liver increased by 63%. Higher lipid peroxidation in the brain could be attributed to higher lipid content, especially in the white matter, which is rich in myelin with lower poly unsaturated fatty acid than grey matter (Svennerholm, 1968). The midbrain exhibited a minuscule increase in lipid peroxidation, possibly due to it being heavily myelinated, thus posed to be more resistant towards oxidative stress. The GSH concentration level was lower in the liver by 31% as compared to the brain. Because of the compromised redox balance, GSH depletion causes the brain to be more susceptible to oxidative stress (Baek et al., 1999).

The animal model conducted by Ullah et al. used CCl4 to induce oxidative stress, which led to acute and chronic liver injury in the mice (Ullah et al., 2020). Male albino mice (BALB/c) weighing between 28 and 34 g were separated into three groups; as the control mice group, experimental and treatment groups. In the experimental group, 2 mL/kg body weight of CCl4 was administered via IP, which caused liver injury in the mice. For the control group, normal saline, which was dissolved in 2% dimethylsulfoxide (DMSO), was administered to the animal model and the treatment group, which were further separated into three different groups, were given three different doses of poncirin (5, 15 and 30 mg/kg) to identify whether the poncirin could reduce the damage caused by CCl4 administration. The administration of CCl4 in the mice significantly increased the NO production in both plasma and liver, thus activating the inflammatory process following liver injury. The CCl4-treated group significantly reduced the antioxidant enzymes such as GSH, GST, Catalase and SOD. Furthermore, an increase in lipid peroxidation was also seen in the CCl4-treated animal group, which was is determined to be due to an increase in MDA (end product of lipid peroxidation) level (Hickma et al., 2004).

The following animal model study was conducted by (Ammar et al., 2022), which used 130–150 g body weight of Wistar rats (Ammar et al., 2022). The Wistar rats were separated randomly into five groups: control, experimental and treatment groups. The experimental group was administered CCl4 with a dose of 2.8 mL/kg subcutaneously. The CCl4 was used in the experiment to induce hepatic and renal injuries in the rats’ models. For the control group, 2.8 mL/kg of olive oil was administered subcutaneously, followed by 0.5 mL/150 g of normal saline with gaps of 6 h from the olive oil administration. Treatment groups received 50 mg/kg of silymarin or naringenin (20 or 40 mg/kg) orally in the treated rats group. This animal model was done for 3 days before the results were obtained. The CCl4-treated rats significantly increased AST and ALT levels compared to the control group, which indicated the liver damage. The AST and ALT enzymes leak from the damaged hepatocytes into the blood, which shows that infiltration of cellular occurs as well as centrilobular necrosis and ballooning degeneration (Ramaiah, 2007). The animal model uses the CCl4 targeting different mechanisms to induce oxidative stress. (Wang et al., 1996). used the animal model of acatalasemic mice, which has less CAT activity as compared to control mice but no difference in GPx activity (Wang et al., 1996). As the CAT level is reduced in the acatalasemic mouse, CCl4 induce-liver injury by increasing the H_2_O_2_ level in the cellular or tissue level. (Ritesh et al., 2015)) animal model induces oxidative stress targeting the brain (Ritesh et al., 2015). The CCl4-treated Wistar rats induced lipid peroxidation and reduced the GSH concentration, thus increased ROS and oxidative stress. The summary of CCl4 research is shown in Table 3.

**TABLE 3:** carbon tetrachloride (CCl4) induced oxidative stress animal model.

Animal model	Experimental animal	Duration	Dose/Amount	Targeted cell/organ	Outcome	Conclusion	References
Carbon tetrachloride (CCl4)	Acatalasemic mice	18 and 24 4	0.0125 mL of CCl4	Liver	Increase in AST and ALT as well as MDA level which indicate liver damage	Oxidative stress increase when AST, ALT and MDA level increase thus leading to liver damage	Wang et al. (1996)
	Wistar rat	24 4	1 mL/kg of CCl4 and sunflower oil	Brain, liver	CCl4 causes a significant decrease in antioxidant activity and an increase in lipid peroxidation in the brain	CCl4-induce oxidative stress in the brain regions by marked alteration of antioxidant balance	Ritesh et al., 2015
	BALB/c mice	7 days	2 mL/kg of CCl4, 50 mg/kg of silymarin, 5 mg/kg of poncirin, 15 mg/kg of poncirin, 30 mg/kg of poncirin	Liver	CCl4 increases lipid peroxidation in the liver and reduces the antioxidant function	Administration of CCl4 increases lipid peroxidation which leads to liver injury in the BALB/c mice	Ullah et al. (2020)
	Wistar rats	3 days	2.8 mL/kgof CCl4, 2.8 mL/kg of olive oil, 20 mg/kg of naringenin, 40 mg/kg of naringenin, and 50 mg/kg of silymarin	Kidney and liver	AST and ALT levels significantly increase in CCl4-injected mice	CCl4 administration can induce oxidative stress in the liver which leads to an increase in AST and ALT levels	Ammar et al. (2022)

Abbreviations: AST, aspartate transaminase; ALT, alanine transaminase; MDA, malondialdehyde.

### 5.3 Diquat (1,1′- ethylene-2,2′-bipyridinium)

Diquat, also known as bipyridyl herbicide, is used to control aquatic weeds and broad-leaved weeds among fruit and vegetables. Diquat is a potent redox cycler that, when reacted with oxygen molecules, can produce superoxide anion (O2•–) and hydrogen peroxide (H_2_O_2_), the cytotoxic mechanism that diquat induces. A high dose of diquat via injection causes liver necrosis, lung injury, and death in rats and mice, which is caused by acute oxidative stress generation of diquat (Wu et al., 2012).

The study conducted by (Zhang et al., 2016) was done to identify the effect of diquat on the female reproductive system (Zhang et al., 2016). Female ICR mice were used and divided into three different groups randomly. The first group served as control mice group and was given was given normal saline, the second group was treated with 8 mg/kg of diquat, and the third group was treated with 12 mg/kg. The mice were injected IP twice a week for four consecutive weeks. The diquat dose was chosen due to previous reports indicating acute toxicity is induced by IP injection of 24 mg/kg body weight (Fu et al., 1999). The variation in the ovary weight was measured to identify the effect of diquat in diquat-treated mice (8 and 12 mg/kg), showed a significant decrease compared to the control mice. Diquat-treated mice had less percentage of normal germinal vesicle (GV) oocytes than control mice. For the first time, this study showed that chronic diquat exposure could reduce the oocyte quality as well as affect early embryo development, which is consistent with the previous report stated the malformations in diquat-treated mallard embryos (Sewalk et al., 2000). Oocytes and embryos are extremely sensitive to ROS, and high ROS levels can disturb oocyte quality and affect the early development of embryos (Zhang et al., 2021). Chronic diquat exposure caused a significant increase in lipid peroxidation, thus increasing the level of MDA in the serum.

A study by (Gupta et al., 2000) used Fischer 334 rats (male with a weight between 180 and 220 g and female with a weight between 160 and 180 g) which were separated into two groups (Gupta et al., 2000). The diquat-treated rats were treated with 0.075–0.25 mmol/kg of diquat, while the control group only received normal saline via IP injection. An increase in ALT level indicated that administration of diquat led to acute hepatic necrosis in diquat-treated Fischer 334 rats. However, a higher dose of diquat was required in female rats to increase the ALT level. An increase in ALT level did not occur in female rats with doses below 0.2 mmol/kg, while in male rats, the increase of ALT level could be observed with a dose of 0.1 mmol/kg of diquat. Diquat-treated female rats produced greater 2,4-dinitrophenylhydrazine (DNPH)-reactive modified protein in liver and bile as compared to diquat-treated male rats which induced cell death.

The animal model study by (Zhang et al., 2021) used ICR mice, which were divided into four groups, control group, treatment group (Vitamin D3), experiment group (diquat and combination of vitamin D3 and diquat) (Zhang et al., 2021). The experiment group for diquat-induced mice was given 10 mg/kg of diquat suspended in 200 µL of phosphate-buffered saline (PBS), administered via IP. The ALT and AST serum levels in diquat-treated mice were significantly higher than in other groups (control, treated and combination of diquat and vitamin D3). The increase in ALT and AST levels indicated that the administration of diquat could decrease the liver function of the mice. The ALT and AST which are mainly synthesized in the liver are important transaminases; hepatocyte damage causes the release of this enzyme into the blood (Pirimoğlu et al., 2020).

The next animal model study was conducted by Cao et al. which used 9.6 kg of piglets and was separated into two groups (control and experiment) (Cao et al., 2018). The experiment group was administered 10 mg/kg of diquat intraperitoneally which the dose was chosen due to previous reports indicating 10 mg/kg of diquat cause oxidative stress in piglets (Lv et al., 2012)^.^ The diquat-treated piglet showed decreased SOD and GSH-Px (Plasma Glutathione peroxidase) activities and increased MDA levels specifically in jejunal mucosa. This result was consistent with previous reports, which showed the effect of diquat administration to an increase in oxidative stress. The advantage of using diquat to induce oxidative stress is the accumulation of diquat in the kidneys and liver relatively than paraquat, which occurs in the lungs. Diquat has lower activation energy than paraquat, so redox cycling is more readily in diquat than paraquat and requires a lower dose to induce oxidative stress (Magalhã et al., 2018). The summary of Diquat’s research is shown in Table 4.

**TABLE 4:** Diquat (1,1′- ethylene-2,2′-bipyridinium) induced oxidative stress animal model.

Animal model	Experimental animal	Duration	Dose/Amount	Targeted cell/organ	Outcome	Conclusion	References
Diquat (1,1′- ethylene- 2,2′- bipyridinium)	ICR mice	4 weeks	8 mg/kg of diquat, 12 mg/kg of diquat, and normal saline	Reproductive system, embryo	Administration of diquat cause a significant reduction of antioxidants (T- SOD, GPx and CAT) and an increase in MDA level	Diquat induces oxidative stress in the ICR mice, which effect the fetus’s development	Zhang et al. (2016)
	Fischer 334 rats	2, 4, or 6 h	0.075–0.25 mmol/kg of diquat	Liver	Diquat increases the ALT level	Oxidative stress increase in diquat- treated rats with an increase in ALT which induces oxidative stress and leads to acute hepatic necrosis	Gupta et al. (2000)
	ICR mice	-	10 mg/kg of diquat	Liver	Significant increase of ALT and AST levels in diquat- treated mice	An increase in AST and ALT levels leads to an increase in oxidative stress and decrease the liver function of the mice	Zhang et al. (2021)
	Piglet	7 days	10 mg/kg of diquat	Intestine	Significant decrease in antioxidant activity (SOD and GSH-Px) and increase in MDA level in diquat-treated piglets	Increase of oxidative stress in diquat-treated piglets, which disturbed the intestinal function and barrier	Cao et al. (2018)

Abbreviations: ICR, institute of cancer research; T-SOD, superoxide dismutase; GPx, Glutathione peroxidase; CAT, catalase; MDA, malondialdehyde; ALT, aspartate transaminase; ALT, alanine transaminase; GSH-Px, Plasma Glutathione peroxidase.

### 5.4 Paraquat (1,1’ -dimethyl-4,4′- bipyridinium dichloride)

Paraquat has been widely used as a broad-spectrum herbicide and is considered harmful to humans and laboratory animals. The paraquat chemical structure is similar to dopaminergic neurotoxin, N-methyl-4-phenylpridinium ion (MPP+) which is the active metabolite of 1-methyl-4-phenyl-1,2,3,6-tetrahydropyridine (MPTP) which cause permanent symptom of Parkinson’s disease (Castello et al., 2007). Exposure to paraquat can induce pulmonary toxicity which has been demonstrated in previous studies that showed retention of paraquat occurs higher in the lung than in other organs (Sharp et al., 1972). Paraquat is considered under the redox cycling class due to its ability to induce mitochondrial damage, ROS production and oxidative stress (Castello et al., 2007). The oxidation of paraquat by oxygen molecule produces superoxide anion radical (O2•–), which can promote lipid peroxidation (Bus and Gibson, 1984). Paraquat radicals can produce H_2_O_2_ which may lead to the formation of more RO radicals, thus being able to damage the cell more severely.

There are several animal models which use paraquat to induce oxidative stress. Long-Evans hooded rats were used during these studies which were separated into a control group (saline injection) and an experimental group (Somayajulu-Niţu et al., 2009). About 10 mg/kg of paraquat dose was administered intraperitoneally on weekly basis for 3 consecutive weeks. The level of GSH in the experimental group was reduced to half as compared to control group. The experimental group has a significant increase in MDA level which is the product of lipid peroxidation. An increase in lipid peroxidation could affect the properties of the membrane and cellular homeostasis leading to apoptosis (Yadav et al., 2019).

The next study using male Swiss albino mice weighing 24 to 30 were randomly separated into paraquat-treated groups and control mice group. The paraquat-treated group was administered 10 mg/kg of paraquat via intraperitoneally injection twice a week for 28 days while the control group received the same volume of 0.9% sodium chloride (NaCl) via IP injection (Sengupta et al., 2017). The previous study identified the sub-lethal dose for paraquat in Swiss albino mice (Mitra et al., 2011). Paraquat-treated mice group has higher ROS generation as compared to the control group which can be seen via Dichlorofluorescein (DCF) fluorescence intensity. In this study, the paraquat produces oxidative stress in the spleen which can be seen in the paraquat-treated group. In the treated group, the GSH level, SOD and CAT activities can decrease compared to the control group, thus explaining the increased oxidative stress in the spleen (Sengupta et al., 2017). The paraquat-induced ROS involves the p53-AKT which causes the splenocyte’s death.

The animal study by McCormack et al. used C57BL/6 mice as the animal model to induce oxidative stress using paraquat (McCormack et al., 2005). The mice were separated into a control group and were administered normal saline, and an experimental group. A 10 mg/kg paraquat dose was injected into the mice intraperitoneally once a week for three weekly injections. This regimen and dose do not induce overt toxicity or pathology in tissues other than the brain. The 4-hydroxy-2-nonenal (4-HNE) increased in the paraquat-induce mice as compared to control group, indicated the neurodegenerative occurred in the experimental group. The 4-HNE is the breakdown product of the decomposition of polyunsaturated fatty acid peroxides that can be used as the biomarkers of lipid peroxidation and to detect oxidative stress damage (Hashimoto et al., 2003).

Based on the above studies observations, we conclude that Paraquat is the most used in vertebrate species to induce oxidative stress due to its toxic effects on humans’ accidental exposure, which is well known. However, the problem arises if the administration of paraquat toward the bird or other non-model species requires a more specific dose to induce the oxidative stress and to account for non-target effects such as accumulation in the lungs or toxicity, which may occur at the low dose (Koch and Hill, 2017). The summary of Paraquat’s research is shown in Table 5.

**TABLE 5:** Paraquat (1,1′- dimethyl- 4,4′- bipyridinium dichloride) induced oxidative stress animal model.

Animal model	Experimental animal	Duration	Dose/Amount	Targeted cell/organ	Outcome	Conclusion	References
Paraquat (1,1′- dimethyl- 4,4′- bipyridinium dichloride)	Long-Evans hooded rats	3 weeks	10 mg/kg of paraquat	Brain	Alteration of lipid peroxidation and GSH level in paraquat-treated rats	Reduce in GSH level and an increase in lipid peroxidation can be seen in paraquat-treated rats, which cause an increase in oxidative stress thus affects brain membrane and cellular homeostasis leading to apoptosis	Somayajulu- Niţu et al. (2009)
	Swiss albino mice	28 days	10 mg/kg of paraquat	Spleen	Decrease in antioxidant activity (GSH, SOD and CAT) in the paraquat-treated mice	Significant increase in oxidative stress in the spleen which involves the p53- AKT pathway lead to splenocyte deaths	Sengupta et al. (2017)
	C57BL/6 mice	3 weeks	10 mg/kg of paraquat	Brain	The lipid peroxidation in paraquat-treated mice increases	Paraquat-induced oxidative stress leads to neurodegenerative	McCormack et al. (2005)

Abbreviations: GSH, glutathione; SOD, superoxide dismutase; CAT, (Catalase); AKT, aspartate transaminase.

### 5.5 Tert-butyl-hydroperoxide (tBHP)

**FIGURE 4:**
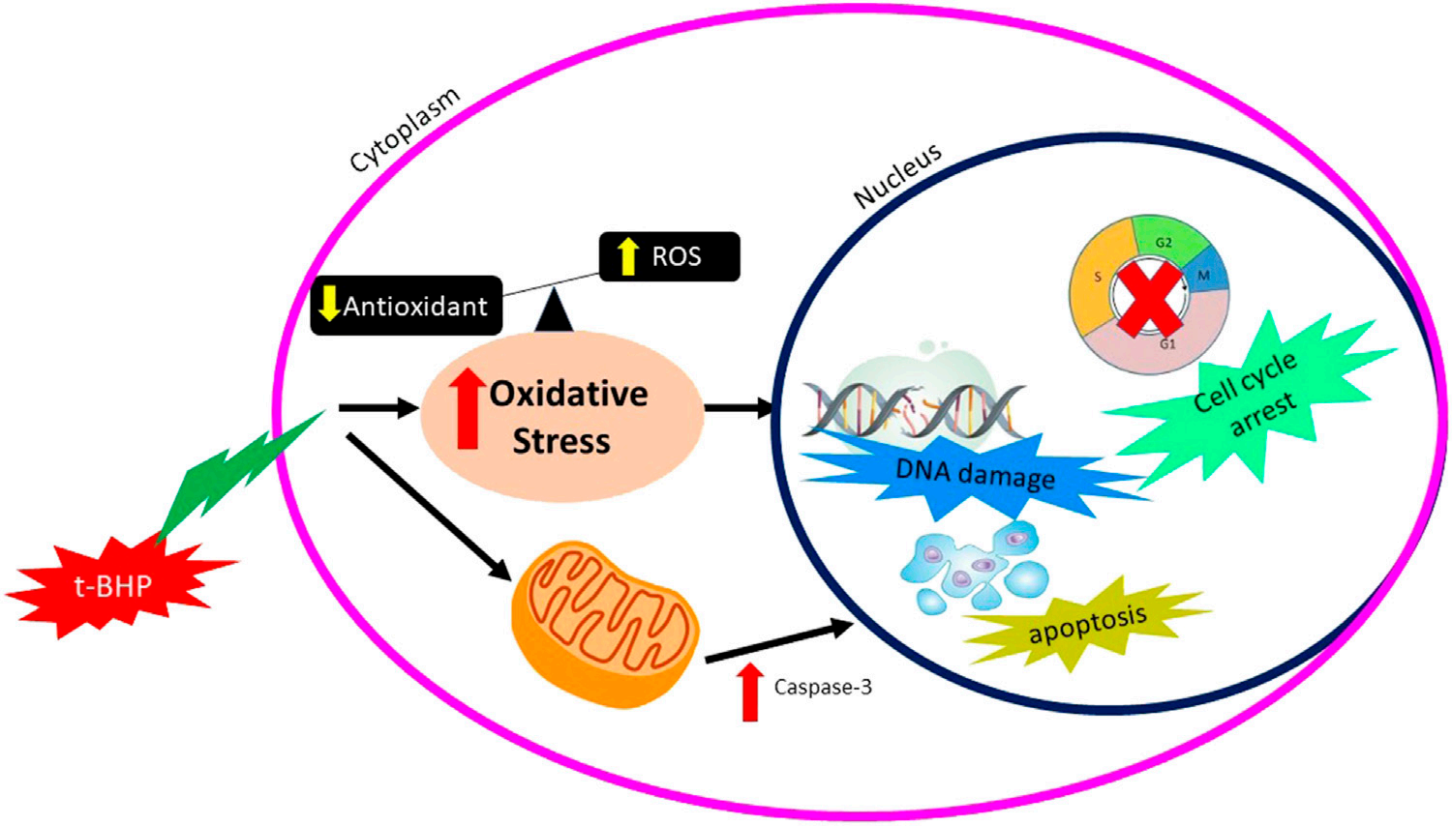
Mechanisms of action of tBHP producing oxidative stress.

Tert-butyl-hydroperoxide (tBHP) is a chemical with an unstable form of H_2_O_2_ metabolized within the cell to induce the oxidative stress effect. A low concentration of tBHP induced caspase-dependent apoptosis and increased the production of ROS. Production of ROS was derived from NADPH oxidase and mitochondria, leading to the tBHP-induced apoptosis. Production of oxidative stress by tBHP can be seen in Figure 4.

*In vitro* study by (Zhao et al., 2017) was done to identify the effect of tBHP on the human umbilical vein endothelial cell (HUVECs) using two different concentrations of tBHP, which are 50 and 500 µm (tBHPH) (Zhao et al., 2017). The tBHPL induced apoptosis and increased ROS production by NADPH oxidase. Overexpression of NOX4 leads to increased ROS generation and may induce endothelial cell death or apoptosis. The tBHPH induced necroptosis and increased ROS production by mitochondria. tBHPH caused the release of lactate dehydrogenase (LDH), which occurs when the plasma membrane is damaged, leading to cell death (Chan et al., 2013). The tBHP-induced apoptosis due to ROS-activated the p38 mitogen-activated protein kinase (p38MAPK) pathway (Yang et al., 2012).

Another study done by (Wu et al., 2020) used adult male Sprague Dawley rats which were divided into two different groups randomly (Wu et al., 2020). The experimental group was administered 300 μmol/kg body weight of tBHP and the control group was administered saline once a day for 15 days via intraperitoneally. The sample was collected at 3, 6, and 9 weeks after the end of treatment. tBHP-treated rats had high lipid peroxidation which could be observed in caput and cauda epididymis at 6 and 9 weeks as compared to their control group. Elevated lipid peroxidation causes tissue damage and cell death. Epididymal spermatozoa of tBHP-treated rats had significant DNA oxidation, damaged sperm DNA. The resu; tant damaged DNA could impact the sperm quality, thus ultimately leads to the infertility (Bansal and Bilaspuri, 2011)^.^ The cellular effect of tBHP was comparable to H_2_O_2_ to induce oxidative stress with the logistical advantage of tBHP, which is chemically stable until it is administered into the cells or tissue (Slamenova et al., 2013). The summary of tBHP research is shown in Table 6.

**TABLE 6:** Tert-butyl-hydroperoxide (*t*BHP) induced oxidative stress animal model.

Animal model	Experimental animal/cell line culture	Duration	Dose/Amount used	Targeted cell/organ	Outcome	Conclusion	References
Tert-butyl- hydroperoxide (*t*BHP)	Human umbilical vein endothelial cells (HUVECs)	1, 2, or 12 h	50 μm of *t*BHP and 500 μm of *t*BHP	Plasma membrane	*t*BHPL induced apoptosis while *t*BHPH necroptosis	Different doses of *t*BHP induce different oxidative stress damage toward the cell	Zhao et al. (2017)
	Sprague Dawley rats	3, 6, and 9 weeks	300 μm oles/kg of *t*BHP	Sperm	Significant increase of lipid peroxidation in *t*BHP-treated rats	*t*BHP induces oxidative stress which damages the sperm DNA and lead to infertility	Wu et al. (2020)

Abbreviations: HUVECs, Human Umbilical Vein Endothelial Cells; t-BHP, tert-butyl hydroperoxide.

## 6 Conclusion and future perspective

ROS influences both disease and normal bodily functions. Every cell function, from growth to diversification and termination, is influenced by ROS. Depending on their levels, they can activate or deactivate various cellular signals. These ROS-driven regulations contribute to tissue development, sustenance, and healing. A complex interplay between antioxidant processes and metabolic activities manages and determines the effects of ROS. Therefore, in reaction to external factors, cells utilize ROS cues to determine their fate. Since oxidative stress indicators manifest early in the course of the development of many illnesses, oxidative stress is an early event in their etiology. ROS are extremely powerful weapons against the majority of biomolecules due to their strong chemical reactivity. In molecular and cell biology research, oxidants are involved in a growing variety of diverse and dynamic activities, often without a clear understanding of the underlying chemical pathways. Our goal was to provide a strong mechanistic foundation by connecting chemistry and biology. In understanding the mechanism of these highly reactive molecules and their function as mediators of oxidative changes in cellular components, we hope that this overview will be useful to researchers.

Antioxidant-rich foods, on the other hand, have shown potential health benefits in reducing oxidative stress and chronic diseases (Bjelakovic et al., 2012). However, its interesting to note that in the past, antioxidant medications were frequently ineffective, and in some cases, they may have even worsened mortality under specific situations. The most common examples of antioxidant interventions are Vitamin A, E, and beta-carotene. The potential risks associated with these conventional antioxidant supplementation caused an increase in mortality rate (Bjelakovic et al., 2007). The incidence of prostate and colorectal cancer has been recorded among morbidity cases following vitamin E therapy (Albanes et al., 1995), whereas, in another study, the combination use of beta-carotene and vitamin A therapies altered lung cancer mortality rates, although without any chemopreventive effect (Omenn et al., 1996).

This suggests that simply increasing dietary antioxidant intake may not be effective in reducing oxidative damage. Instead, researchers believe that modulating the body’s own antioxidant levels through the use of weak pro-oxidants could be a more promising strategy for managing diseases associated with reactive oxygen species. This approach focuses on enhancing the body’s natural defense mechanisms rather than relying solely on external sources of antioxidants (Halliwell, 2013). Further research is needed to understand the specific mechanisms and optimal dosages of antioxidants for prevention and treatment. Additionally, exploring alternative approaches such as lifestyle modifications and a balanced diet can be beneficial for overall health and wellbeing (Bjelakovic et al., 2012).

Furthermore, due to the potential for beneficial effects, more attention needs to be paid to examining the pathophysiology, epigenetic markers, and animal models that trigger oxidative stress studied in this review. The animal model for oxidative stress has been used to demonstrate several perspectives on the effects of oxidative stress. However, additional research on the reagent’s utilization in animal models is necessary to determine the precise doses—both the lowest and maximum—that can be employed to induce oxidative stress. By altering the mechanism of ROS via inhibiting or minimizing oxidative stress, the insights on animal models presented in this study offer future perspectives on the potential biomarkers and therapeutic options. Henceforth, the drug’s intervention strategies could be investigated further in treating disease following the underlying mechanism and biomarkers discussed here. We will strive further to highlight and document in detail about antioxidant therapeutic interventions with least adverse effects in our future review study.
